# Protocol for the culturing of primary hippocampal mouse neurons for functional *in vitro* studies

**DOI:** 10.1016/j.xpro.2024.102991

**Published:** 2024-04-11

**Authors:** Teresa M.L. Cramer, Shiva K. Tyagarajan

**Affiliations:** 1 University of Zurich, Institute of Pharmacology and Toxicology, Winterthurerstrasse 190, 8057 Zurich, Switzerland

**Keywords:** Cell Biology, Neuroscience

## Abstract

Primary hippocampal cultures grown from genetically modified mice provide a simplified context to study molecular mechanisms underlying neuronal development, synaptogenesis, and synapse plasticity *in vitro*. Here, we describe a simple protocol for culturing hippocampal neurons from P0 to P2 mice and a strategy for inducing alterations in synaptic strength at inhibitory and excitatory synapses *in vitro.* We also describe approaches for immunofluorescent labeling, image acquisition, and quantification of synaptic proteins.

For complete details on the use and execution of this protocol, please refer to Cramer et al.^1^

## Highlights

• Precise culturing and manipulation of primary hippocampal neurons from mice
• Induce and quantify synaptic plasticity in cultured mouse hippocampal neurons
• Techniques for labeling, image acquisition, and quantification of synaptic proteins
• Versatile framework to study synaptic plasticity, neuronal development, or disorders

## Abstract (editor highlights)

Publisher’s note: Undertaking any experimental protocol requires adherence to local institutional guidelines for laboratory safety and ethics.

## Graphical abstract

**Figure:**
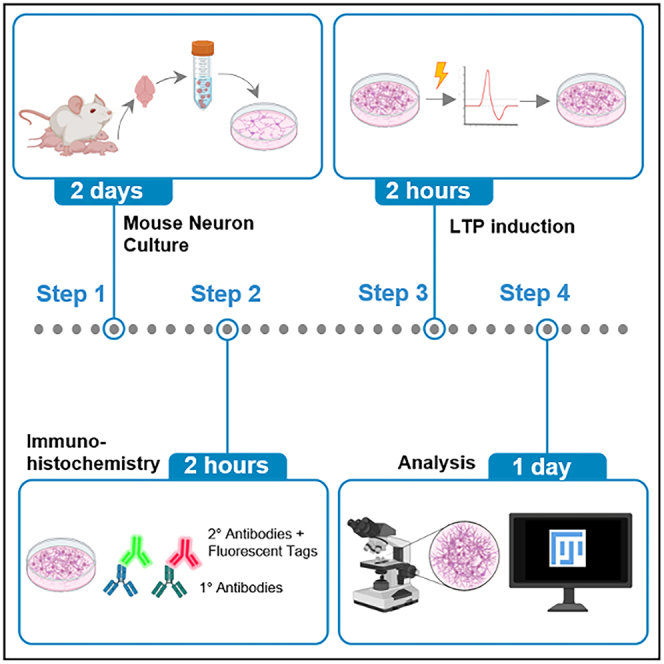

## Before you begin

The protocol below describes the specific steps for culturing, maintaining, and genetically manipulating primary hippocampal neurons grown from mice. Resultant cultures exhibit reproducible properties, including excitatory/inhibitory neuron ratios, which enable the investigation of mechanisms underlying synaptic plasticity. As changes in the expression of synaptic proteins *in vitro* are important correlates of neuronal plasticity *in vivo*, this protocol also describes the specific steps for inducing plasticity in cultured hippocampal neurons, as well as immunofluorescent labeling, image acquisition, and quantification approaches. This protocol is based on an initial publication investigating the function of Adamtsl3 in inhibitory and excitatory synaptic plasticity using knock-out cultures grown from *Adamtsl3*^*flox/flox*^ mice.^1^ The presented protocol, however, can easily be applied in various settings. Before you begin, you will need to complete the following preparatory steps.

### Institutional permissions (if applicable)

All the procedures in animal studies must be approved by the Institutional Animal Care and Use Committee according to established regulations and guidelines. In this protocol, all procedures were reviewed by the European Community Council Directives of November 24, 1986 (86/609/EEC) and approved by the cantonal veterinary office of Zurich.

### Breeding preparations

**Timing: 19 ± 1 day**

Primary neurons should be grown from P0-P2 pups. When breeding mice for primary neuron preparation, it is advantageous to breed two females with one male to increase the number of pups born and thereby, the yield of neurons. When mice are set up for breeding, it is also advantageous to check the female after 1 day of mating for the vaginal plug to estimate the birth date (females will deliver in 19 ± 1 day after plug) and plan accordingly for the neuron preparation. In this protocol, we crossed mice to breed pups homozygous for the floxed allele of *Adamtsl3*.

### Adeno-associated virus (AAV) selection for transduction

AAVs are commonly used for gene delivery in primary neuron cultures, as AAV particles lead to high expression of the transgene in > 90% of neurons (depending on the promoter close to 100%).^2^ The AAV serotype and promoter used for the transgene should be considered when selecting an AAV for transduction. We selected an AAV8-hSyn1-RFP-Cre (AAV8-hSyn1-RFP control) due to the high neuronal tropism of AAV serotype 8 and hSyn1 promoter conferring neuron-specific long-term transgene expression.^3^^,^^4^

**Caution:** All work using viruses must be performed in agreement with your institution’s biosafety committee and appropriate biosafety level.

### Material and reagent preparation for mouse neuron culture

The following materials and reagents should be prepared earliest 2 days before the preparation of primary neuron cultures. Sterilize all solutions and disinfect any equipment used in this protocol with either 70% ethanol or if possible, by autoclaving.

The following materials are sufficient to prepare mouse neurons from up to 6 pups and culture 24 coverslips (60,000–70,000 neurons per coverslip). If preparing cultures from more pups or different genotypes in parallel, the number of reagents and aliquots has to be changed accordingly.

### Prepare coverslip

**Timing: 30 min**

Primary hippocampal neurons are plated and maintained on 18 mm Poly-L-Lysine coated coverslips. The coverslips are washed and maintained in an incubator before use.

**Figure 1:**
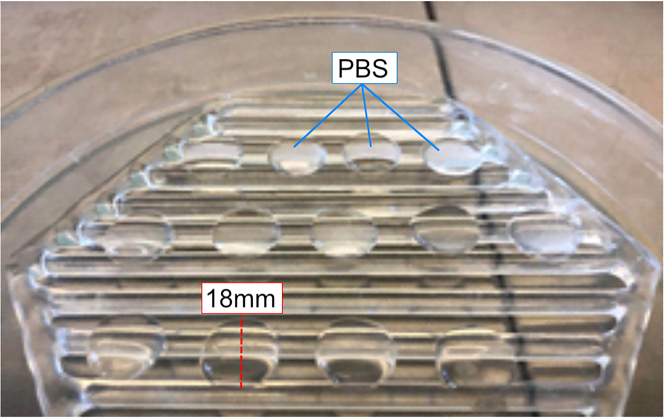
Preparation of coverslips Wash coverslips four 4 times with PBS. Leave the last PBS rinse on coverslips and incubate 12–16 h at 37°C.

***Note:*** This step can be performed 1–2 days before mouse brain dissection, not earlier.

***Note:*** Coverslips coated manually with Poly-L-Lysine (Sigma, Cat no. P-6282) may be equally effective. For this, dilute Poly-L-Lysine stock to a final concentration of 100 μg/mL in sterile sodium borate buffer (150 mM, pH 8.4). Pipette 200 μL Poly-L-Lysine / sodium borate solution onto each coverslip and incubate 12–16 h in the incubator at 37°C and 5% CO_2_. Rinse 4× with sterile PBS and leave the last PBS wash to sit on coverslips in the incubator until used.

1. Using sterile tweezers, arrange 24 glass coverslips coated with poly-L-lysine on a sterile glass rack.
2. Wash 4 times with sterile PBS (250 μL per coverslip) and aspirate completely each time.
  a. Leave the last PBS wash to sit on coverslips as shown in Figure 1 and place in the incubator at 37°C and 5% CO_2_ until used.

**CRITICAL:** All steps should be performed under sterile conditions in a laminar flow hood using sterilized tools. If possible, sterilize by autoclaving.

### Prepare media and reagents

**Timing: 2–4 h (as needed)**

3. Prepare the required media for the preparation of primary hippocampal neuron cultures (see materials and equipment below).

## Key resources table

**Table:** 

REAGENT or RESOURCE	SOURCE	IDENTIFIER
**Antibodies**		

Rabbit polyclonal anti-Adamtsl3 (dilution 1:1,000)	Home-made	Cramer et al.^1^
Mouse monoclonal anti-gephyrin (dilution 1:1,000)	Synaptic Systems	Cat no. 147 111
Mouse monoclonal anti-PSD95 (dilution 1:1,000)	NeuroMap	Cat no. 73-028
Guinea pig monoclonal anti-VGAT (dilution 1:1,000)	Synaptic Systems	Cat no. 131 004
Guinea pig polyclonal anti-VGLUT (dilution 1:1,000)	Synaptic Systems	Cat no. 135 304

**Other**		

CLSM 800 Airyscan microscope, Carl Zeiss		

**Bacterial and virus strains**		

AAV8- hSyn1-RFP-Cre	Viral Vector Facility, UZH/ETH	Cat no. v230
AAV8- hSyn1-RFP	Viral Vector Facility, UZH/ETH	Cat no. v133

**Chemicals, peptides, and recombinant proteins**		

Glucose	Sigma	Cat no. G8270
B-27 Plus neuronal culture system	Thermo Fisher Scientific	Cat no. A3653401
L-glutamine	Gibco	Cat no. 25030-032
Gentamicin	Gibco	Cat no. 15710-049
Amphotericin B	Gibco	Cat no. 15290-018
FCS	Gibco	Cat no. 20270-106
BSA	Sigma	Cat no. A-9418
Papain	Sigma	Cat no. P-4762
Poly-L-lysine-coated coverslips (18 mm)	Neuvitro Corporation	Cat no. GG-18-PLL
DNase I	Sigma	Cat no. 10104159001
Hilgenberg Pasteur pipettes	Thermo Fisher Scientific	Cat no. 10534131
NGS	Jackson Laboratory	Cat no. JAC005-000-121
CNQX	Tocris	Cat no. 0190
Tetrodotoxin	Sigma	Cat no. 4368-28-9
(N-methyl-D-aspartic acid) NMDA	Sigma	Cat no. 6384-92-5
D-APV	Tocris	Cat no. 0106
Strychnine	Sigma	Cat no. 57-24-9
Bicuculline	Tocris	Cat no. 0130
Na_2_HPO_4_ anhydrous	Sigma	Cat no. 71640
NaH_2_PO_4_ monohydrate	Axon Lab	Cat no. 1227KG001

**Deposited data**		

Synaptic cluster analysis (Python-script ImageJ)	Cramer et al.^1^	https://github.com/dcolam/Cluster-Analysis-Plugin

**Experimental model: Organism/strain**		

Mouse: *Adamtsl3*^flox/flox^ BL6		Both male and female P0-P3 pups were used in the experiments.

## Materials and equipment

### Reagents for mouse neuron preparation

**Table:** PBG Solution – Papain Dissolvent

Reagent	Final concentration	Amount
PBS	N/A	49.5 mL
BSA	1 mg/mL	50 mg
Glucose	10 mM	0.5 mL
**Total**	**N/A**	**50 mL**

Aliquot and store at −20°C for up to 6 months. Avoid freeze-thaw cycles.

**Table:** PBS/Glucose – Dissection Solution

Reagent	Final concentration	Amount
PBS	N/A	497.25 mL
Glucose	5.5 mM	2.75 mL
**Total**	**N/A**	**500 mL**

Filter-sterilize (pore size: 0.22 μm) before usage and store at 4°C for up to 2 weeks.

**Table:** Plating Medium

Reagent	Final concentration	Amount
Neurobasal Plus	N/A	17.2 mL
B27	0.02%	0.4 mL
FCS	0.1%	2 mL
Amphotericin B	50 μg	0.2 mL
Gentamycin	2 mM	0.2 mL
**Total**	**N/A**	**20 mL**

Filter-sterilize (pore size: 0.22 μm) and store at 4°C for up to 2 weeks.

**Table:** Culture Medium

Reagent	Final concentration	Amount
Neurobasal Plus	N/A	194 mL
B27	0.02%	4 mL
L-glutamine	2 mM	2 mL
**Total**	**N/A**	**200 mL**

Filter-sterilize (pore size: 0.22 μm) and store at 4°C for up to 2 weeks.

### Reagents for iLTP & eLTP induction

**Table:** iLTP Control Solution

Reagent	Stock	Final concentration	Amount
Water (deionized)	**N/A**	**N/A**	36.95 mL
NaCl	1 M	145 mM	7.25 mL
KCl	1 M	2 mM	0.1 mL
HEPES	1 M	10 mM	0.5 mL
CaCl_2_	1 M	2 mM	0.1 mL
MgCl_2_	1 M	2 mM	0.1 mL
Glucose	1 M	10 mM	5 mL
**Total**	**N/A**	**N/A**	**50 mL**

Filter-sterilize (pore size: 0.22 μm) and store at 4°C. Prepare on the day of the experiment.

**Table:** iLTP Induction Solution

Reagent	Stock	Final concentration	Amount
Water (deionized)	**N/A**	**N/A**	36.95 mL
NaCl	1 M	145 mM	7.25 mL
KCl	1 M	2 mM	0.1 mL
HEPES	1 M	10 mM	0.5 mL
CaCl_2_	1 M	2 mM	0.1 mL
MgCl_2_	1 M	2 mM	0.1 mL
Glucose	1 M	10 mM	5 mL
NMDA	10 mM	20 μM	200 μL
CNQX	10 mM	10 μM	100 μL
**Total**	**N/A**	**N/A**	**50 mL**

Filter-sterilize (pore size: 0.22 μm) and store at 4°C. Prepare on the day of the experiment.

**Table:** eLTP Control Solution

Reagent	Stock	Final concentration	Amount
Water	**N/A**	**N/A**	40.551 mL
NaCl	1 M	125 mM	6.25 mL
KCl	1 M	2.5 mM	0.125 mL
MgCl2	1 M	1 mM	0.05 mL
CaCl2	1 M	2 mM	0.1 mL
Glucose	1 M	33 mM	1.65 mL
HEPES	1 M	5 mM	0.25 mL
D-APV	100 mM	20 μM	0.0095 mL
Strychnine	5 mM	3 μM	0.03 mL
Bicuculline	100 mM	20 μM	0.0095 mL
Tetrodotoxin	1 mM	0.5 μM	0.025 mL
**Total**	**N/A**	**N/A**	**50 mL**

Filter-sterilize (pore size: 0.22 μm) and store at 4°C. Prepare on the day of the experiment.

**Table:** eLTP Induction Solution

Reagent	Stock	Final concentration	Amount
Water	**N/A**	**N/A**	40.551 mL
NaCl	1 M	125 mM	6.25 mL
KCl	1 M	2.5 mM	0.125 mL
CaCl2	1 M	2 mM	0.1 mL
D-Glucose	1 M	33 mM	1.65 mL
HEPES	1 M	5 mM	0.25 mL
Strychnine	5 mM	3 μM	0.03 mL
Bicuculline	100 mM	20 μM	0.0095 mL
Glycine	10 mM	200 μM	0.95 mL
**Total**	**N/A**	**N/A**	**50 mL**

Filter-sterilize (pore size: 0.22 μm) and store at 4°C. Prepare on the day of experiment

**CRITICAL:** Exposure to D-APV, bicuculline, strychnine, or tetrodotoxin may result in serious health effects. Handle in agreement with your institution’s biosafety committee and appropriate biosafety level.

### Reagents for immunofluorescence

**Table:** Na-phosphate buffer

Reagent	Final concentration	Amount
Water (distilled)	N/A	800 mL
Na_2_HPO_4_ anhydr.	N/A	46.0 g
NaH_2_PO_4_ monohydrate	N/A	10.5 g
Adjust to 1000 mL with distilled water		
**Total**	**N/A**	**1000 mL**

Adjust pH using 1 M NaOH 1 M HCl if pH is < 7.2 and > 7.4. Store at 25°C–30°C for up to 6 months. Before use mix well by stirring.

**Table:** 4% PFA Fixative

Reagent	Final concentration	Amount
Paraformaldehyde	4%	8 g
Water plus 4 drops of NaOH (8 N)	N/A	125 mL
Na-phosphate buffer	0.15 M	75 mL
**Total**	**N/A**	**200 mL**

Aliquot and store at −20°C for up to 3 months. Avoid freeze-thaw cycles.

**CRITICAL:** Exposure to paraformaldehyde can result in serious health effects. Handle in agreement with your institution’s biosafety committee and appropriate biosafety level.

## Step-by-step method details

This section describes a simple and fast protocol for culturing primary hippocampal neurons from P0 to P2 mice. In addition, we describe a strategy for inducing alterations in synaptic strength at excitatory and inhibitory synapses to explore mechanisms underlying synaptic plasticity *in vitro.*

### Prepare fire-polished Pasteur pipettes for dissociation

**Timing: 5 min**

This step describes how to fire-polish Pasteur pipettes to dissociate digested mouse brain tissue by shear force.

**Figure 2:**
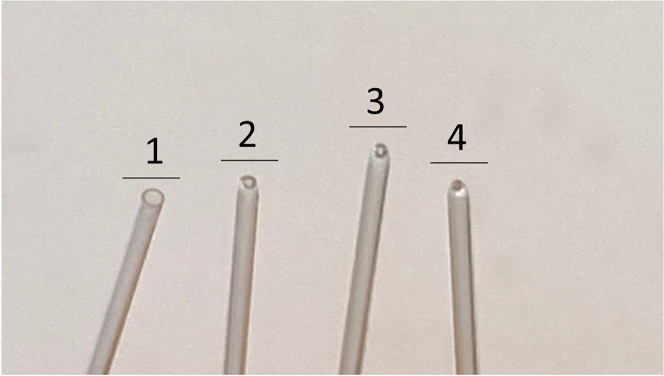
Preparation of fire-polished Pasteur pipettes Round out the tip of a Pasteur pipette to produce 3 sizes: (1) original tip diameter (2) ∼70% of original diameter (3) 45% of the original diameter and (4) ∼15% of original diameter.

1. Fire-polish glass Pasteur pipettes of successively smaller diameter.
  a. Light a Bunsen burner.
  b. Attach a bulb to the pipette.
  c. Heat the tip of a sterile Pasteur pipette over the burner flame by passing it quickly (2–3 times) through the flame to decrease the tip diameter.
    ***Note:*** Continuously squeeze and release the bulb as airflow through the pipette will help prevent the tip from closing up completely.
  d. Examine the tip: Ensure the hole is still open and the edges of the tip are rounded out.
  e. Examine the diameter of the tip: Produce 3 pipettes with gradually decreasing outer diameter: ∼70%, to ∼15% of the original opening as shown in Figure 2.
    ***Note:*** Repeat Step 1c as many times as necessary to achieve the desired diameter size with round edges.
    ***Note:*** Other dissociation methods such as the Miltenyi gentle MACS dissociator (Miltenyi, Cat no. 130-093-235) may be equally effective.^5^

### Prepare papain solution

**Timing: 5 min**

This step describes how to dissolve papain for the digestion of mouse brain tissue and should be set up immediately before mouse brain dissection.

2. Dissolve papain in 5 mL PBG to a final concentration of 0.5 mg/mL.
  a. Heat to 37°C in a water bath for 40–60 min.
  b. Store at 25°C–30°C until use.

***Note:*** Adjust the volume if necessary (5 mL of papain/PBG is sufficient for 5–6 hippocampi).

### Dissect mouse brain

**Timing: 5 h**

This step describes how to isolate the hippocampi from P0-P2 mouse pups from both male and female sex.

3. Inside a sterilized laminar flow hood, prepare your sterilized dissecting tools: one big scissor, one small micro-dissecting scissor, 2 pairs of micro-dissecting fine forceps (tip size 2 mm), and a fine spatula with flat rounded ends.

***Note:*** We recommend using a big scissor such as Sigma, Cat no. Z265993, a small scissor such as Sigma, Cat no. S3146, 2 pairs of fine forceps such as Sigma, Cat no. F3767 and a spatula such as Sigma, Cat no. S9147. However, equivalent dissecting tools are equally effective.

4. Inside the hood, fill one 100 mm and six 60 mm Petri dishes half full with cold PBS/glucose (one to collect the brains and six to dissect each hippocampi) and place them on ice.
5. Inside the hood, fill a 15 mL Falcon tube with 2 mL cold PBS/glucose to collect hippocampi and place on ice.
6. If possible, place a binocular microscope in the laminar flow hood.

***Note:*** If not possible, dissection steps can be performed on a clean bench.

7. Prepare an additional ice bucket and place dry task wipes on top of it.

***Note:*** Task wipes serve to protect the pup’s skin from the ice during anesthesia.

8. Transfer 1–2 pups onto the cold task wipes to induce hypothermia anesthesia.
  a. Monitor for lack of movement and after 2–3 min, confirm anesthesia by very gently squeezing a paw.
  b. Sacrifice the deeply anesthetized pup by cervical dislocation.
  c. Decapitate the pup.
  d. Isolate the brain in the 100 mm petri dish filled with PBS/glucose on ice in the laminar flow hood (refer to Figure 3).
    i. Stabilize the brain using a pair of forceps pierced through the eyes.
    ii. Cut the skull from the lateral edges using another pair of forceps, starting from the nose region.
    iii. Cut the skull between the eyes. Lift the bones of the skull to unfold the brain.

**Figure 3:**
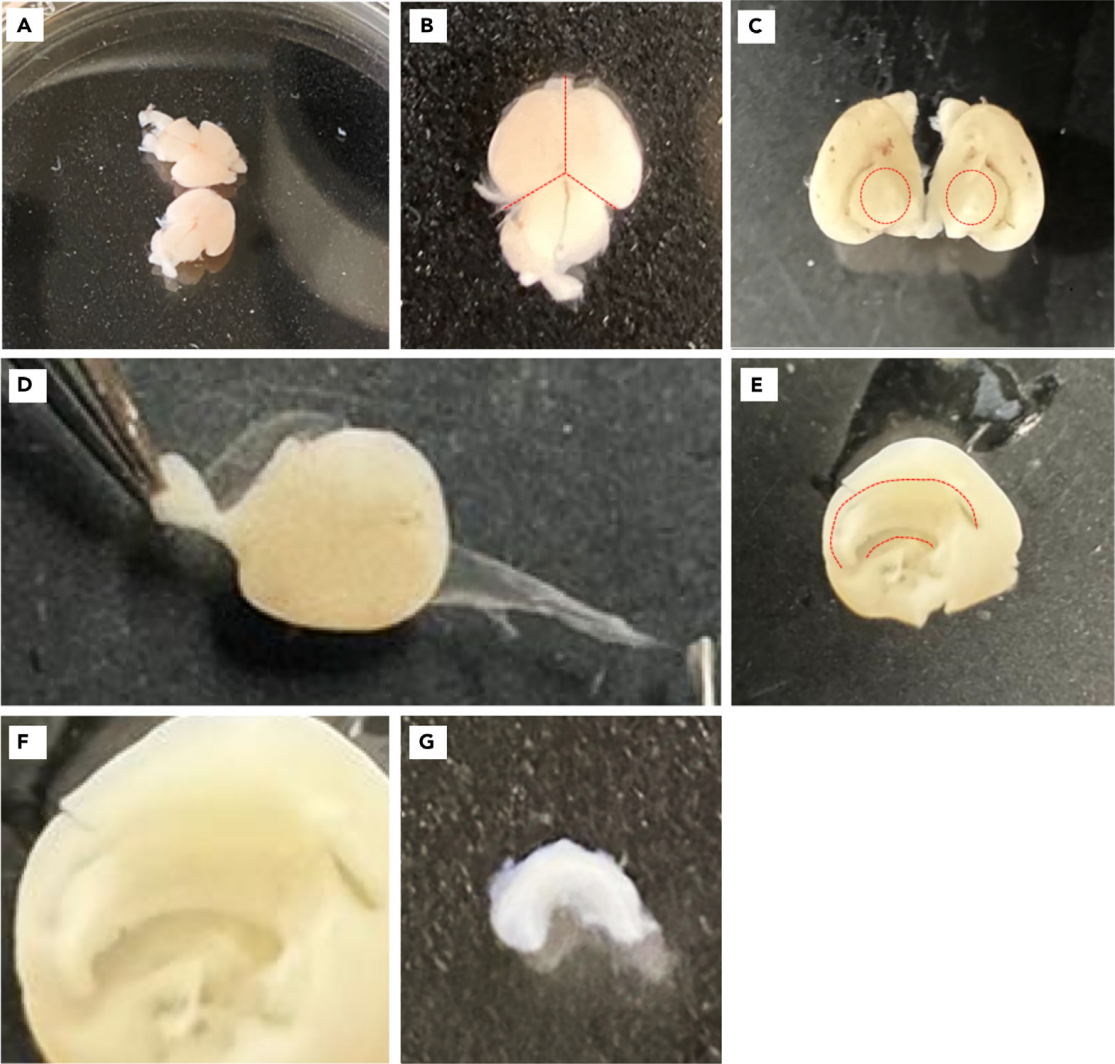
Isolation of mouse brain and hippocampi (A) Isolated P0 mouse brains. (B) Dissect mouse brains at the dotted line to separate the two hemispheres and remove the brainstem. (C) Remove any brain tissue below the telencephalon indicated with the dotted circles. (D) Hold the hemisphere by the olfactory bulb and start pulling the meninges from the dorsal end of the cortex. (E) The hippocampus is highlighted by the dotted lines (C-shaped). (F) Zoom in of the hippocampus. (G) Isolated hippocampus.
  e. Transfer the brain to a clean 60 mm dish filled with PBS/glucose as shown in Figure 3A and place it under the microscope.
9. Remove the meninges.
  a. Hold the brain at the brainstem with 1 pair of forceps.
  b. Carefully separate the two hemispheres and remove the brainstem by cutting along the dotted line shown in Figure 3B using the other pair of forceps.
  c. Carefully remove any brain tissue below the telencephalon on each hemisphere indicated with the dotted circle in Figure 3C.
  d. Flip the hemisphere so that the cortex is facing outwards. Hold the hemisphere by the olfactory bulb and start pulling the meninges from the dorsal end of the cortex as shown in Figure 3D.
    ***Note:*** The meninges can be distinguished by reddish color.
    ***Note:*** Try to grasp a large piece of the meninges; the larger the piece, the higher the chances that you can pull it off in its entirety.
  e. Flip the hemisphere again and remove all meninges from the interior part.
10. Isolate the hippocampi.
  a. Arrange the hemispheres so that the interior is facing up. The cortex is rolled inwards to form the hippocampus, which appears like a bulged C-shaped structure attached to the cortex fold (indicated by the dotted lines in Figure 3E, zoom in Figure 3F).
  b. Isolate the hippocampus by ‘unrolling’ the cortex fold and cut along the edge of the dorsal hippocampi border (higher dotted line in Figure 3E).
  c. Repeat this step on the other hemisphere.
  d. Collect the hippocampi as shown in Figure 3G in a clean 15 mL Falcon tube containing 2 mL PBS/glucose.
11. Repeat Steps 8–10 until all hippocampi have been isolated and pool them in 15 mL Falcon tube.

### Dissociate and plate mouse neurons

**Timing: 4 h**

In this step, hippocampi are digested, dissociated, and subsequently plated on coverslips.

12. Sterilize papain/PBG solution by filtration using a 0.22 μm filter.
  a. Add DNase I to a final concentration of 10 μg/mL.
13. Carefully aspirate as much PBS/glucose as possible from the 15 mL Falcon tube without damaging the hippocampi.
14. Add papain/PBG/DNase I solution to hippocampi and incubate for 15 min at 37°C.
  a. Every 5 min, gently tap the Falcon tube to mix the content.
15. Carefully aspirate the papain/PBG/DNase I solution and wash 2 times with 5 mL plating medium at 25°C–30°C.
16. Add 2 mL of plating medium.
17. Mechanically dissociate neurons with the fire-polished Pasteur pipette until the suspension is homogeneous.
  a. Start with the largest diameter Pasteur pipette (5 up and down).
  b. Continue with a medium-diameter Pasteur pipette (5 up and down).
  c. Finish with the smallest diameter Pasteur pipette (∼2 up and down).

***Note:*** Repel neurons against the wall of the Falcon tube to facilitate dissociation.

***Note:*** Adjust movements depending on homogeneity.

18. Count cells in a Neubauer chamber using Trypan blue.
  a. Dilute cells to the desired end volume in plating medium (60,000–70,000 cells/200 μL).

***Note:*** Two hippocampi correspond to about 500,000 cells/mL.

19. Remove the coverslips from the incubator and aspirate the PBS from the coverslips.
  a. Add 200 μL of dissociated cell suspension to each coverslip as shown in Figure 4.

**Figure 4:**
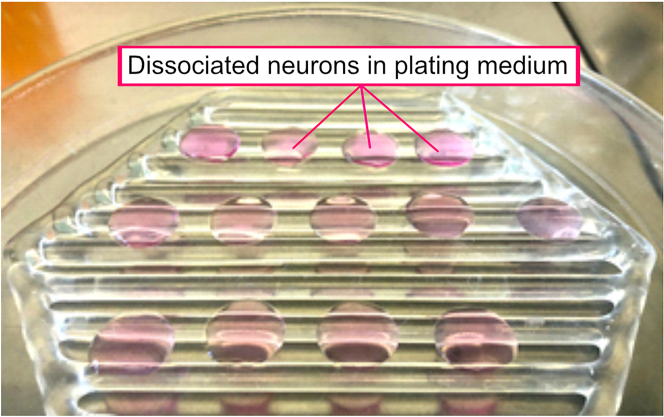
Plating of dissociated neurons onto coverslips Remove PBS from coverslips and seed neurons in a plating medium on the coverslips for 2–3 h at 37°C.
  b. Incubate at 37°C and 5% CO_2_ for 2–3 h.
20. Fill a 12 well-plate with 2 mL of culture medium per well and incubate at 37°C.
  a. After 2–3 h, transfer the coverslips into the warm 12 well-plate.
  b. Incubate at 37°C and 5% CO_2_.

### Infect mouse neurons with AAV

**Timing: 30 min**

This step involves the transduction of primary neuron cultures with an AAV expressing a transgene of interest.

We used an AAV8-hSyn1-RFP-Cre (AAV8-hSyn1-RFP control) to achieve Cre-dependent deletion of Adamtsl3 in cultures grown from *Adamtsl3*^*flox/flox*^ pups.

21. At 4 days-in-vitro (DIV), infect the cultures with AAV.
  a. Transfer 1/2 of the conditioned medium from each well of the cell culture plate into a sterilized Eppendorf tube.
  b. Add 1 μL of AAV8-hSyn1-RFP to half of the Eppendorf tubes and AAV8-hSyn1-RFP-Cre to the other Eppendorf tubes.
  c. Gently mix the Eppendorf tubes by pipetting up and down.
  d. Add the conditioned media containing AAV back into the corresponding well.
  e. Incubate at 37°C and 5% CO_2_.

***Note:*** 1 μL of an AAV titer of 1.0 × 10^13^ viral genome (vg) /ml – 5.0 × 10^12^ vg/ml is sufficient to achieve 100% infection. AAV genome titers tend to be provided by the manufacturer but can be confirmed by qPCR using a plasmid DNA standard as described by Aurnhammer, Christine et al.^6^ Adjust AAV infection volume as required.

### Maintain mouse neuron cultures

**Timing: 1 h spread over 5–7 days**

This step involves the maintenance of mouse neuron cultures for up to 16 days.

22. After 6 DIV start monitoring the color of the medium and cell survival under the microscope.
  a. Change 1/3 to 1/2 of the medium if it appears yellowish (After 6–8 DIV, media change every 2–3 days is usually needed).

***Note:*** Primary neuronal cultures are very fragile. Minimize the time neuron cultures spend outside the incubator.

### Induce synaptic plasticity

**Timing: 2 h**

In this step, excitatory and inhibitory forms of long-term potentiation (LTP) are induced in mature mouse neuron cultures.

23. At 14 DIV induce inhibitory LTP (iLTP) in half of the coverslips.
  a. Arrange three clean 12 well plates next to each other in a hood.
    i. Label the columns. Example: 3 times RFP-Control, 3 times Cre-Control, 3 times RFP-iLTP, 3 times Cre-iLTP.
    ii. Fill the first and last rows of all plates with warm iLTP control solution per (1 mL/well).
    iii. Fill the second row of 1 ½ plates with warm iLTP induction solution (1 mL/well).
  b. Transfer 6 coverslips infected with AAV8-hSyn1-RFP and 6 coverslips infected with AAV8-hSyn1-RFP-Cre into the first rows filled with iLTP control solution.
    i. Let incubate for 10–15 min at 37°C and 5% CO_2_.
  c. Transfer 3 coverslips infected with AAV8-hSyn1-RFP and 3 coverslips infected with AAV8-hSyn1-RFP-Cre into the second row filled with iLTP induction solution.
    i. Let incubate for 2 min at 25°C–30°C.
    ii. Transfer all coverslips (from the first or second row) into the last row containing the iLTP control solution.
    iii. Let incubate for 60 min at 37°C and 5% CO_2_.

***Note:*** This protocol was adapted from Petrini et al.^7^

24. Induce excitatory LTP (eLTP) in half of the coverslips.
  a. Arrange three clean 12 well plates next to each other in a hood.
    i. Label the columns. Example: 3 times RFP-Control, 3 times Cre-Control, 3 times RFP-eLTP, 3 times Cre-eLTP.
    ii. Fill the first and last rows of all plates with warm eLTP control solution (1 mL/well).
    iii. Fill the second row of 1 ½ plates with warm eLTP induction solution (1 mL/well).
  b. Transfer 6 coverslips infected with AAV8-hSyn1-RFP and 6 coverslips infected with AAV8-hSyn1-RFP-Cre into the first rows filled with eLTP control solution.
    i. Let incubate for 20 min at 37°C and 5% CO_2_.
  c. Transfer 3 coverslips infected with AAV8-hSyn1-RFP and 3 coverslips infected with AAV8-hSyn1-RFP-Cre into the second row filled with eLTP induction solution.
    i. Let incubate for 10 min at 25°C–30°C.
    ii. Transfer all coverslips (from the first or second row) into the last row containing the eLTP control solution.
    iii. Let incubate for 40 min at 37°C and 5% CO_2_.

***Note:*** This protocol was adapted from McLeod et al.^8^

### Immunofluorescence of coverslips

**Timing: 4–5 h**

This step involves the immunofluorescent labeling of synaptic proteins to be subsequently visualized by confocal microscopy.

Labeling should be performed 60 min after iLTP induction and 40 min after eLTP induction.

**Figure 5:**
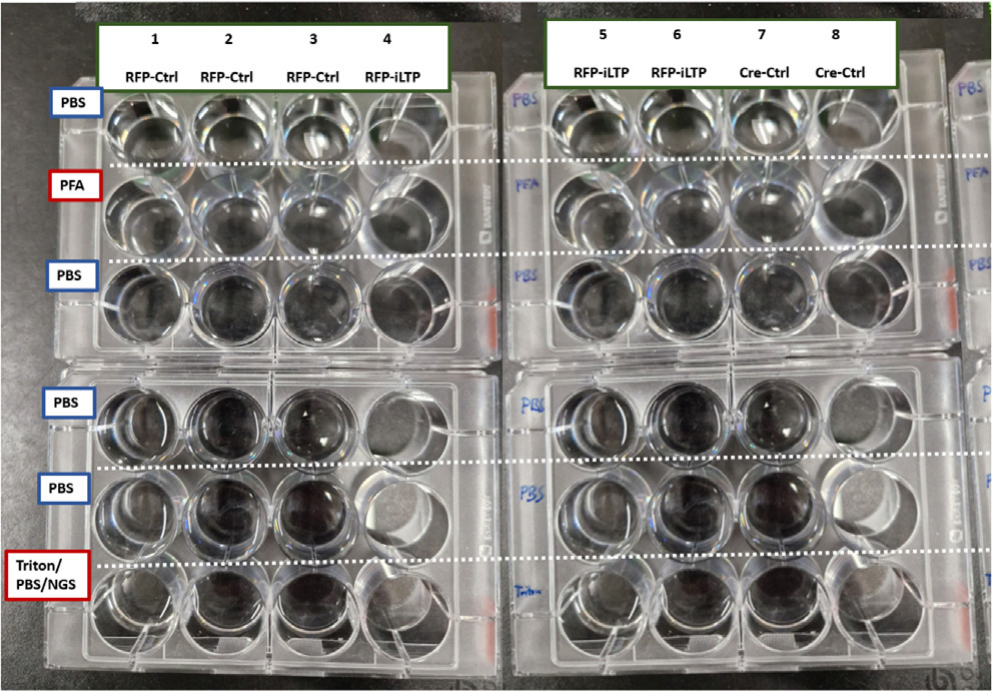
Preparation of 12-well plates for washing, fixing, and penetrating neuron cultures on coverslips before immunofluorescence
  b. Label the columns. Example: 3 times RFP-Control, 3 times RFP-iLTP, 3 times Cre-Control, 3 times Cre-iLTP.
  c. Fill the first rows with PBS to the top.
  d. Fill the second row with 1 mL 4% PFA fixative per well.
  e. Fill the third, fourth and fifth row with PBS to the top.
  f. Fill the last row with 1 mL 0.2% Triton X-100 solution in PBS with 10% NGS for permeabilization as shown in Figure 5.
26. Dilute 1° and 2° antibodies in 1.8 mL PBS with 10% NGS as blocking agent (plan 150 μL/coverslip) and keep on ice or at 4°C.

***Note:*** Appropriate antibody targets to evaluate the expression of iLTP include the γ2 subunit of GABA_A_ receptors, VGAT and gephyrin.

25. During 60 min incubation of iLTP, prepare for the immunofluorescent labeling of coverslips.
  a. Arrange 6 clean 12 well plates three by two on a clean surface (see example in Figure 5).

**Table:** Antibody mix example

**1° antibody solution**	

Antibody	Dilution
Guinea pig monoclonal anti-VGAT	1:1000
Mouse polyclonal anti-Gephyrin	1:1000

**2° Antibody solution**	

Antibody	Dilution
Guinea pig Alexa Fluor 647	1:500
Mouse Alexa Fluor 488	1:500

***Note:*** 1° Antibody concentrations are suggested by the manufacturer and tend to be very high. 1:1000 is a good starting point.

27. 60 min after iLTP induction, gently rinse the coverslips by dipping them into the first row of 12 well plates filled with PBS.
  a. Place coverslips facing up into the second row containing 4% PFA.
    i. Fix neurons for 10–15 min.
  b. Wash the coverslips by dipping them into rows 3, 4, and 5 filled with PBS.
  c. Place coverslips facing up into 0.2% Triton X-100 solution in PBS.
    i. Permeabilize the neurons for 3–5 min.

***Note:*** In most cases, no additional permeabilization steps are needed. If results indicate insufficient labeling of intracellular proteins, Steps 27c and 27i can be extended to 8 min.

**Figure 6:**
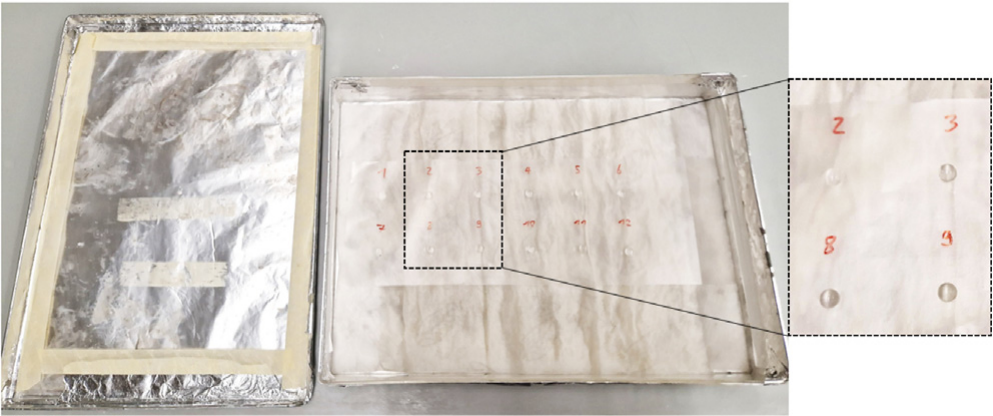
Light-protected antibody incubation chamber Zoom in on the right shows parafilm on wet Kleenex with 120 μL of antibody solution; place coverslips upside down onto the drops of antibody solution.
  b. Place coverslips upside down onto the drops of 1° antibody solution.
    i. Incubate for 90 min in the dark.

***Note:*** In most cases, no additional blocking is needed. If results indicate high background fluorescence, NGS concentration in 1°antibody mice may be increased to 20%.

28. Prepare a wet Kleenex with a Parafilm on top in a large glass Petri dish and label for different samples.
  a. Pipette 120 μL of 1° antibody solution containing NGS blocking agent onto the Parafilm as shown in Figure 6.

29. Transfer coverslips to a 12-well plate filled with PBS.
  a. Wash for 10 min in the first row on a shaker (very slow rotation).
  b. Transfer to the second row and wash for 10 min on a shaker (very slow rotation).
  c. Transfer to the third row and wash for 10 min on a shaker (very slow rotation).
30. Prepare a second wet Kleenex with a Parafilm on top in a large glass Petri dish and label it for different samples.
  a. Pipette 120 μL of 2° antibody solution containing NGS blocking agent on the Parafilm.
  b. Place coverslips upside down onto the drops of 2° antibody solution.
    i. Incubate for 30 min in the dark.

***Note:*** In most cases, no additional blocking is needed. If results indicate high background fluorescence, NGS concentration in 2°antibody mice may be increased to 20%.

31. Transfer coverslips to a 12-well plate filled with PBS.
  a. Wash coverslips as in Step 27.
32. Transfer coverslips facing up on Kleenex and let dry.
33. Label microscope slides.
  a. Mount coverslips facing upside down with DAKO mounting medium onto the microscope slides.
  b. Let dry at 25°C–30°C in the dark for 24 h.
    i. Move to 4°C in the dark for long-term storage.

***Note:*** Under these storage conditions, we found that coverslips can reliably be re-imaged for up to 12 months. Longer storage may prevent reliable analysis.

34. Repeat the immunofluorescent staining method for coverslips 40 min after eLTP induction.
  a. Use appropriate antibodies.

***Note:*** Appropriate antibody targets to evaluate the expression of eLTP include the GluA1 subunit of AMPA receptors, VGLUT1 and PSD95.

**Table:** Antibody mix example

**1° antibody solution**	

Antibody	Dilution
Guinea pig monoclonal anti-VGLUT1	1:1000
Mouse monoclonal anti-PSD95	1:1000

**2° Antibody solution**	

Antibody	Dilution
Guinea pig Alexa Fluor 647	1:500
Mouse Alexa Fluor 488	1:500

### Imaging coverslips

**Timing: 4–6 h**

This step discusses an approach to image fluorescently-labeled proteins within fixed tissue on coverslips.

We performed near super-resolution microscopy using an Airy scan microscope (CLSM 800 Airyscan, Carl Zeiss). Images were taken using a 40× objective with a numerical aperture of 1.4 and pixel size of 112 nm^2^. A near super-resolution microscopy approach per se is not required, but is beneficial to quantify high puncta density. When using a confocal microscopy approach, higher resolutions can be obtained by optimizing the Z-stack (0.25 micron thick and 8 optical sections) at the scanning speed of 4. Utilizing the frame averaging option also improves the image resolution.

35. Blind yourself to the sample condition (Example: control/iLTP > Group A/Group B) to reduce potential bias.
36. Carefully wipe the slide/coverslip using 70% ethanol and place the coverslip facing down on the inverted microscope.
37. Select the 40× objective and a zoom of 1.5.

***Note:*** Higher resolution imaging using a 63× objective may also be of relevance to some investigators to yield high-quality images.

38. Find an infected neuron.
  a. Place the soma in the corner of the image.
  b. Focus on the proximal dendrite.

***Note:*** Proximal dendrites are defined as the dendritic element located between the initial aspiny portion and the most distal 30 μm of a given dendrite (located ∼51 ± 1 μm from the soma).

39. Determine the excitation line and emission collection range based on the specific fluorescence dyes selected for each channel, including their excitation and emission spectra.
  a. Find the optimal settings for scan speed and frame averaging.
    ***Note:*** Scan speed refers to how many lines of your sample are being scanned per second. As the scan speed increases, the pixel sampling time reduces, which results in a decreased signal-to-noise ratio and reduced image resolution.
    ***Note:*** Frame averaging refers to how many times your image (frame) is scanned; the data for each point is then averaged from all frames obtained to reduce noise, which is why higher frame averaging increases the signal-to-noise ratio.
  b. Adjust the laser power, master gain, and digital offset to achieve maximum resolution and a favorable signal-to-noise ratio.
    ***Note:*** For the laser power setting, we recommend starting with the lowest laser power possible; then, laser power can be increased to obtain higher image quality. For most lasers, we do not recommend power settings above 5% to avoid photobleaching.
    ***Note:*** The master gain setting controls the sensitivity of the detector to photons of light emitted from the sample. Increasing gain increases the detection of fluorophores, but also increases noise (background signals) in the resulting image. Balance the gain to obtain an optimal detection of signal to minimal detection of noise.
    ***Note:*** The digital offset setting controls the threshold below which pixels are defined as being black, which allows background/noise to be removed from the final image. Balance the digital offset to obtain an optimal detection of fluorescent signals.
    **CRITICAL:** Keep in mind that these parameters must remain constant for all samples and groups that are intended for quantitative comparison.
    **Caution:** When evaluating the laser power, master gain, and digital offset, regularly check for image saturation. Do not under- or over-saturate the image as this can limit fluorophore detection and mask changes in fluorescence intensity respectively – both may result in data loss when performing analysis. Test for saturation using the Hi-Lo function (press Ctrl-H to select the Hi-Lo LUT). If blue pixels appear (indicating zero intensity) reduce the offset, if red pixels appear (indicating saturation) reduce the laser power.
40. Prepare to take a Z-stack image.
  a. Set the upper and lower limits of the Z-stack based on one fluorescence channel.
    ***Note:*** Keep the number of Z-stacks, step size, and channel selected for setting stack limits constant for all samples that are intended for quantitative comparison.
    ***Note:*** Select a channel for setting the stack limits that are most relevant for your analysis. We chose the 488 channels (gephyrin for iLTP and PSD95 for eLTP) to best detect these post-synaptic markers along the proximal dendrite.
  b. Obtain an image spanning a significant portion of the proximal dendrite height.
    ***Note:*** 8 optical sections with a step size of 0.25 tend to span a significant portion of the proximal dendrite height.
    ***Note:*** Adjust the number of Z-stacks and step size if necessary, based on your specimen (most dendrites in fixed cell culture samples are 2–6 μm thick).
41. Take the image.
  a. Apply Airyscan image processing.
  b. Take images of 15–20 neurons from each coverslip.

***Note:*** Airyscan image processing applies pixel reassignment and deconvolution to the raw images to generate the final super-resolution images.

## Expected outcomes

**Figure 7:**
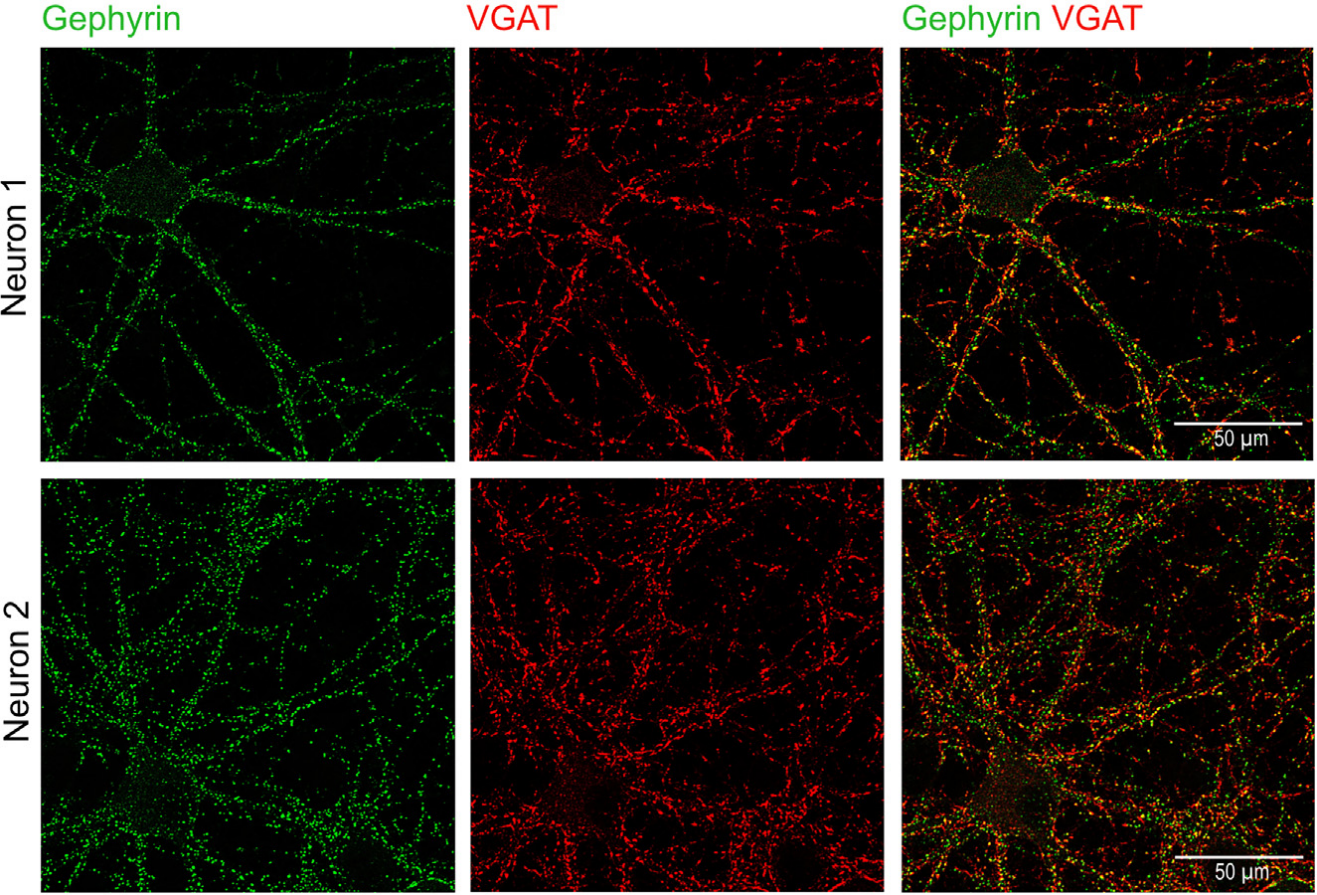
Expected images of neurons acquired and processed using Airy scan microscopy

Images acquired and processed using Airy scan processing can be saved for analysis as LSM or CZI files. We combined the Z-planes into a single image (example shown in Figure 7) using a `maximum projection' (Image > Stacks > Z-project > Projection type: Max Intensity). In the resultant Z-projection immunofluorescence labeled synaptic proteins appear as small blobs or “puncta,” typically less than 1 μm diameter.

Differences in the quantity of synaptic puncta between coverslips may be visible during imaging. At 60 min post-iLTP stimulation, you can expect an increase in gephyrin and VGAT puncta density, as well as increased surface expression of γ2 subunit GABA_A_ receptor. At 40 min post-iLTP stimulation, you can expect an increased puncta density of VGLUT1, PSD95, and surface expression of the GluA1 subunit of AMPA receptors.

***Note:*** For labeling of surface proteins, tissue penetration (Step 25f) should be avoided to exclusively label receptors and/or subunits present on the surface.

## Quantification and statistical analysis

**Timing: 2–3 h**

This step describes a strategy to quantify the abundance, spatial localization, and overlap between fluorescently-labeled proteins in Z-stacked images obtained by high-resolution Airyscan imaging.

We used a custom Python script employing the ImageJ-processing framework.^1^^,^^9^ The script can be used as a plugin and is openly available on a GitHub repository Database: (https://github.com/dcolam/Cluster-Analysis-Plugin).

1. Install and open the plug in.
  a. Plugin > Cluster Analysis > Beta.
2. Select your input folder and set your preferred parameters as shown in Figure 8.
  a. Select “manual selection”.
    ***Note:*** Manual selection allows you to manually choose the area of proximal dendrite you intent to analyze. Automatic selections are beneficial when puncta within a defined channel should be analyzed (Example: DAPI, MAP2, etc.).
  b. Select the channels you want to analyze and enter channel names.
  c. Define the background radius.
    ***Note:*** The background radius should be larger than a typical object in the image. Test using process > Substrate Background > preview.
  d. Define the Gaussian blur.
    ***Note:*** A Gaussian blur of 1 is usually sufficient to smooth an image and reduce noise. Test using Process > Filters > Gaussian blur filter.
  e. Click ok to proceed.

**Figure 8:**
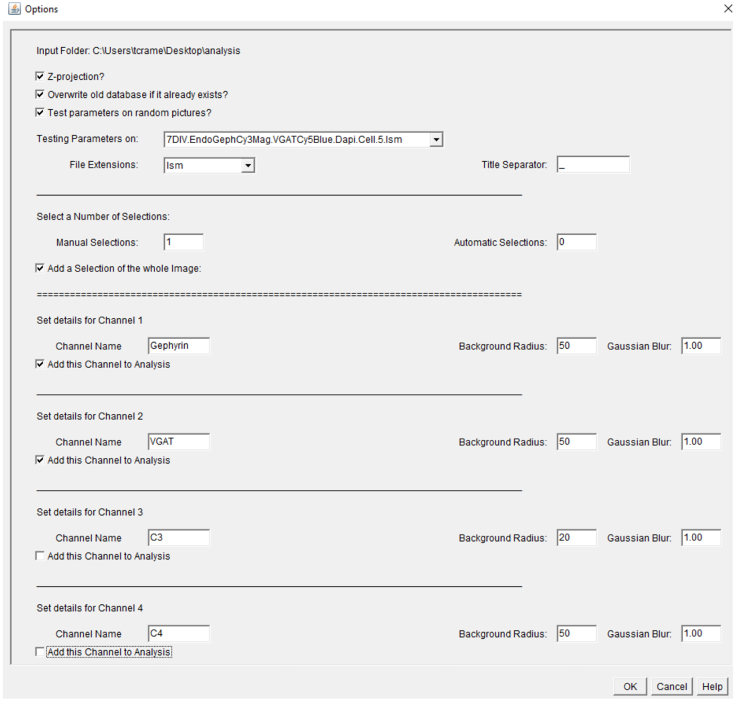
Plugin window for entering analysis parameters described in Step 44
3. Set particle analysis parameters for each channel as shown in Figure 9.
  a. Set your lower and upper particle sizes.
    ***Note:*** A particle size of 0.025–2.5 is adequate to select synaptic puncta.
  b. Set your circulatory range.
    ***Note:*** A range of 1–2 allows detection of all puncta shapes.
  c. Define your threshold method.
    ***Note:*** Thresholding divides an image into two classes of pixels called “foreground” and “background. Foreground pixels retain their original values and are quantified, while background pixels are not. Test using Image > Adjust > Threshold. Otsu and Triangle are effective for synaptic puncta detection.
  d. Click Watershed algorithm.
    ***Note:*** Watershed separation is beneficial for synaptic puncta detection as it increases the detection of individual particles when close together.
  e. Click ok to proceed.

**Figure 9:**
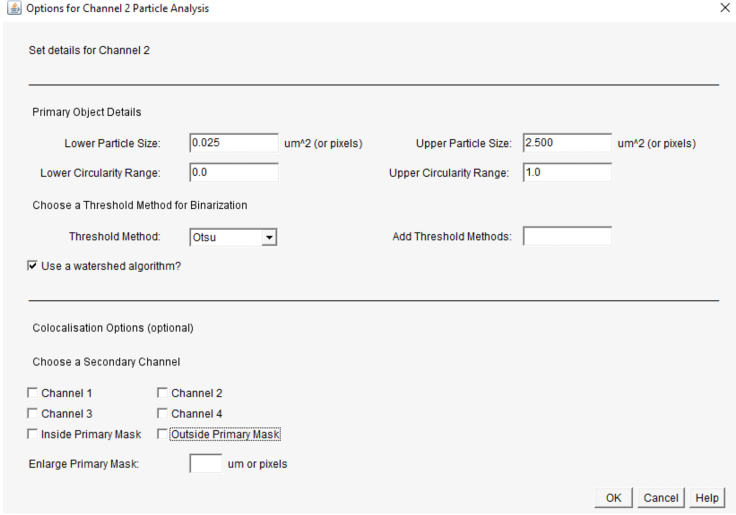
Plugin window for entering analysis parameters for individual channels described in Step 45
4. Enter a name for your manual selection (Example: proximal dendrite).
  a. Save the region of interest (ROI).
    ***Note:*** The “rotated rectangle” selection is ideal for dendrite selection.
  b. Click ok to proceed.
    ***Note:*** Keep the selection area of all proximal dendrites that are intended for quantitative comparison constant.
5. Inspect the result (detected puncta) compared to the original image (example shown in Figure 10).
  a. Click “start experiment” if particle selection is satisfactory.

**Figure 10:**
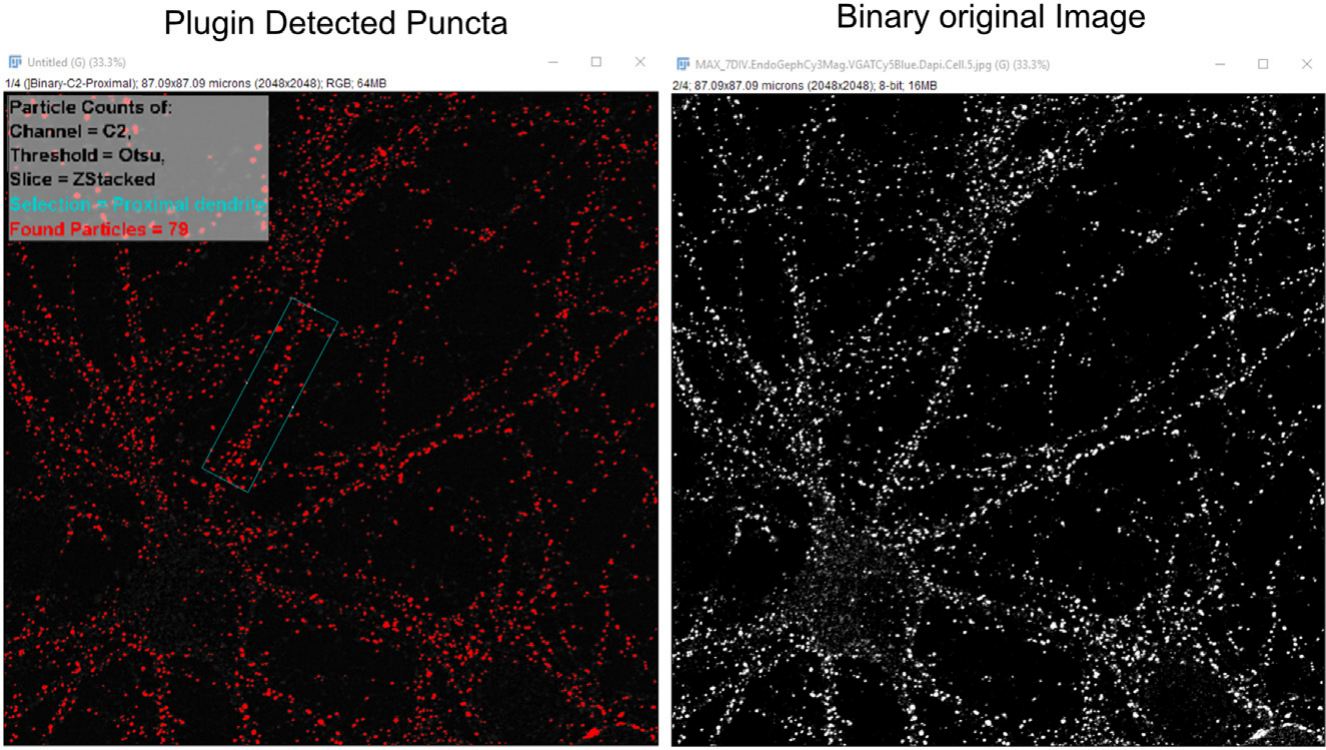
Example plugin detection of immunofluorescently labeled puncta The left side shows puncta detected using plugin. The right side shows the original binary image.

***Note:*** Adjust parameters such as background radius, threshold method, and particle size limits if particle detection is not satisfactory.

6. Analyze your results.
  a. A “Particle Analysis” folder will be saved along with your images.
  b. Go to Particle Analysis > Output Table, and open Particle Analysis Table.
  c. Divide your particle number by the selected proximal dendrite area (puncta density).
  d. Plot “puncta density along proximal dendrite” for Groups A and B.
  e. Unblind yourself.

## Limitations

Our protocol describes primary neuron cultures grown from mouse hippocampi. These cultures should thus, not be used to study cortical or cerebellar neurons. While no adaptations to our protocol are necessary to culture cortical neurons, cerebellar neurons are best cultured at P4-6, when the number of granule cell progenitors peak.^10^ Currently, the maximum duration of the experiment is limited by the health of primary neurons in the culture. In this protocol, the health of primary mouse neurons is optimal for up to 16 days *in vitro*. Thereafter, frequent media changes spur glia growth and contamination.

## Troubleshooting

### Problem 1: Meningeal cell overgrowth

It is of utmost importance to remove the meninges as completely as possible (Step 9). If the meninges is not removed completely, the fibroblast-like phenotype of meningeal cells will overgrow and obscure neurons.

### Potential solution

Curved pre-chilled forceps tend to increase the grip on the meninges. When you peel the meninges off by the olfactory bulb, hold a large enough piece to prevent the breaking of the meninges while pulling it. Do not release it when at the forebrain, but instead turn the tissue over, and carefully peel away remaining meninges on the interior portion of the hemisphere.

### Problem 2: Low transduction efficiency

High AAV transduction efficiency is essential to achieve uniform transgene expression and to observe consistent phenotypes following the induction of synaptic plasticity. Transduction efficiency is influenced by many factors, including the health of neurons, the degree of confluency, and AAV titer (Steps 21 and 22).

### Potential solution

To achieve the highest transduction efficiency, test three different concentrations of AAV in advance. Collect total cell RNA and perform quantitative reverse-transcription polymerase chain reaction (qRT-PCR) in these samples to ascertain the efficiency of transgene expression / or target gene deletion. If AAVs are not available, lentiviral particles can be used, which are equally effective and result in transgene expression in a high percentage of neurons.^11^ Similar to AAV transduction, the expression here depends on the promoter used for the transgene. Transfection of DNA plasmids using Lipofectamine is not recommended, as it only results in transgene expression in a low percentage of neurons (less than 5%).

### Problem 3: Glial cell overgrowth

During the process of culturing primary neurons, neural tissue is dissociated, which activates microglia and may lead to glial cell proliferation. While a small percentage (up to 15%) of glia in culture is important to facilitate neuron survival and function, glial cell overgrowth (>25%) can obscure neurons and alter functional read-outs of neuron manipulations (Step 22).

### Potential solution

When you start monitoring the cultures for neuron health and survival at 6 days *in vitro*, take note of glial cell growth. These can be identified based on their morphology: astrocytes appear star-shaped while oligodendrocytes appear round with spikes around them. To control glial cell proliferation, if necessary, consider antimitotic drugs such as cytarabine furanoside (AraC) up to a concentration of 50 μM and 5-fluoro-2′-deoxyuridine (FUdR) up to a concentration of 25 μM.^12^ As these may have significant neurotoxic effects, do not exceed the indicated dosage. Note: If the meninges are not removed completely before the hippocampus dissociation step, they will proliferate rapidly along with the glial cells. The use of AraC and FUdR will restrict meninges growth, but this will compromise neuron health and imaging quality.

### Problem 4: Contamination

In this protocol, antibiotic and antifungal agents are included in the plating medium, but not in the culture medium. Primary neuronal cultures are therefore highly susceptible to contamination after plating (After Step 20).

### Potential solution

To avoid contamination, confirm that all reagents you are working with are sterile or autoclaved. Additionally, confirm that all equipment you are using, such as the incubator and laminar floor hoods, are properly functioning and thoroughly cleaned with 70% ethanol. Lastly, ensure you are following basic lab rules, including wearing lab coats, gloves and tying back your hair. If contamination occurs and persists, amphotericin B and gentamicin can be added to the culture medium. These, however, could be toxic in cell culture systems and should only be used conservatively.

### Problem 5: Failure to induce synaptic plasticity

Effective induction of synaptic plasticity at inhibitory and excitatory synapses in control neurons (transduced using AAV8-hSyn1-RFP) is essential to obtain reliable read-outs (Steps 23 and 24). Failure to induce synaptic plasticity in control neurons prohibits conclusions from being drawn for experimental neurons (transduced using AAV8-hSyn1-RFP-Cre).

### Potential solution

When the induction of synaptic plasticity fails in control neurons, confirm that reagents for iLTP and eLTP induction have not expired and that the appropriate drug concentrations were used. For any drugs used, avoid freeze-thaw cycles by storing 5–20 μL aliquots in the −20°C freezer (or according to the manufacturer’s recommendations). Neuron health can also affect the induction of synaptic plasticity. Therefore, confirm neuron health before induction and ensure media components have not expired (or stored at −4°C for more than 2 weeks). Sufficiently high neuron density additionally maximizes the likelihood of detecting molecular and morphological changes in neurons. Confirm that cell counting is performed reliably (Step 18) and consider plating neurons at a density of 80,000–90,000 neurons/coverslip (Step 19) if neuron survival appears to affect density during culture maintenance. Electrophysiological LTP induction protocols should not be considered, as they only result in the activation of a small set of synapses.

### Problem 6: Low detection of immunofluorescent-labeled proteins

Reliable immunofluorescence labeling of fixed neuron cultures is essential for subsequent image acquisition, analysis, and extraction of meaningful results (Steps 25–34). Synaptic proteins appear as small blobs or “puncta,” following immunofluorescence. Weak or no immunofluorescent signals can be caused by many factors including antibody incompatibility, fixation, and/or permeabilization method.

### Potential solution

Confirm primary and secondary antibody compatibility (cross-reactivity) as well as antibody dilution. Recommended antibody dilutions including incubation times are usually provided by the manufacturer but should be tested beforehand. Additionally, confirm fixation and permeabilization methods to ensure samples were not over-fixed resulting in antigen masking or insufficiently permeabilized resulting in lack of binding (Step 27). Excessive background signals can be avoided by reducing antibody concentration and increasing washing of coverslips (Steps 29 and 31). Incubation of secondary antibodies and storage of samples should additionally be performed in the dark, as fluorophore signals may fade when exposed to light for an extended period (Steps 30–33).

## Resource availability

### Lead contact

Further information and requests for resources and reagents should be directed to and will be fulfilled by the lead contact, Shiva K. Tyagarajan (shiva.tyagarajan@gmail.com).

### Technical contact

Technical questions on executing this protocol should be directed to and will be answered by the technical contact, Teresa Cramer (teresa.cramer@pharma.uzh.ch).

### Materials availability

All reagents newly generated in this study are available from the lead contact without restriction.

### Data and code availability

• All data reported in this paper will be shared by the lead contact upon request.
• The custom Python script using the ImageJ image-processing framework is openly available on a GitHub repository: https://github.com/PDKlab/Exocytose-EventsDetection. All original code has been deposited at Zenodo and is publicly available. DOIs are listed in the key resources table.
• Any additional information required to utilize the data reported in this work paper is available from the technical contact upon request.
